# Rural-urban inequities in childhood immunisation in Nigeria: The role of community contexts

**DOI:** 10.4102/phcfm.v3i1.238

**Published:** 2011-09-22

**Authors:** Diddy Antai

**Affiliations:** 1 Division of Epidemiology, Institute of Environmental Medicine, Karolinska Institute, Sweden; 2 Division of Global Health & Inequalities, The Angels Trust, Nigeria

## Abstract

**Context:**

Childhood vaccinations are one of the most cost-effective means of reducing negative child health outcomes. Despite the benefits of immunisation, inequities persist both between and within rural-urban areas in Nigeria.

**Objectives:**

To assess the role of community contexts on rural-urban inequities in full immunisation uptake amongst children 12 months of age and older.

**Methods:**

Data from the 2003 Nigeria Demographic and Health Survey including 6029 live born children from 3725 women aged 15–49 years were examined using multilevel regression analysis.

**Results:**

Rural children were disadvantaged both in the proportion receiving full immunisation and individual vaccines. Contextual or community-level factors such as community prenatal care by doctor, community hospital delivery, and region of residence accounted for significant rural-urban inequities in full immunisation.

**Conclusion:**

This study stresses the need for community-level interventions aimed at closing rural-urban inequities in the provision of maternal and child health care services.

## Introduction

### Setting

Vaccinations are effective, inexpensive, and cost-effective means for preventing several infectious diseases.^1^ It is estimated that about 27 million children and 40 million pregnant women do not receive the full complement of vaccines, out of which over 2 million people die worldwide yearly from vaccine preventable diseases.^2^ Vaccine-preventable diseases (VPDs) constitute major causes of morbidity and mortality in Africa. In spite of this, vaccination coverage rates for the various childhood vaccines in Nigeria are amongst the lowest in the world,^3^ with Nigeria being amongst the ten countries worldwide having vaccine coverage below 50%.^4^

Individual, community and systemic factors have been shown to influence the equitable uptake of childhood immunisation in Nigeria and other countries in sub-Saharan Africa.^5^ Whilst much is known about systemic barriers (vaccine supply, distribution, costs, and provider skills)^6, 7^ and individual-level factors (poor understanding of immunisation, suspicions, myths, and rumours,^8^ low maternal education,^9, 10^ maternal employment and working outside the home,^11^ younger maternal age,^9^ delivering away from a health facility and not possessing an immunisation card^12, 13^) that determine immunisation uptake within rural areas of developing countries, such as Nigeria, much less is known about the role of community-level characteristics on rural-urban inequities in childhood immunisation. Nigeria's population is largely rural with more than 53% of the population living in rural areas,^14^ and with the widespread rural-urban inequities in immunisation coverage to the disadvantage of rural children, rural-urban disparities are of particular relevance for immunisation services.

#### Significance of the study

An individual's socio-economic position and health-seeking behaviour are influenced by various infrastructural and institutional characteristics at the community level such as the availability of healthcare services, distance to health care facilities, lack of transportation.^15, 16^ These community-level factors in turn depend on the availability of resources within different community and the broader geographic area, which may decrease or increase the accessibility of healthcare services, such as childhood immunisation uptake within the community.^17, 18^ This study hypothesises that the community contexts in which an individual resides influences the likelihood of childhood immunisation uptake.^19^ On this basis, this study aims to examine the effect of contextual or community-level factors on rural-urban inequities in full immunisation uptake, whilst controlling for individual-level characteristics.

## Ethical considerations

This study was based on secondary data with all participant identifiers removed. Survey procedures and instruments were approved by the National Ethics Committee in the Federal Ministry of Health, Nigeria and by the Ethics Committee of the Opinion Research Corporation Macro International Incorporated (ORC Macro Inc.), Calverton, USA. Ethical permission for use of the data in the present study was obtained from ORC Macro Inc.

## Methods

### Data collection

Data for the study were obtained from the 2003 Nigeria Demographic Survey (DHS). This is a nationally-representative probability sample, collected using a stratified two-stage cluster sampling procedure, according to the list of enumeration areas developed from the 1991 Population Census sampling frame. Initial sampling involved the selection of 365 clusters or primary sampling units (PSUs) with a probability proportional to the size. Subsequent sampling involved systematically selecting households from the previously selected clusters, resulting in a sample of 7864 households. Data were collected by face-to-face interviews from the 3725 women aged 15–49 years in these households. These women contributed a total of 6029 live born children born to the survey. Information collected included birth histories, in-depth demographic and socio-economic information on illnesses, medical care, immunisations, and anthropometric details of children.^20^ Immunisation status of a child was determined from vaccination cards shown to the DHS interviewer. In the absence of vaccination cards, mothers were asked to recall whether the child had received BCG, polio, DPT (including the number of doses for each) and measles vaccinations. How was recall bias handled?

### Measurements

#### Outcome

The outcome variable is the likelihood of a child 12 months of age and older having received all of the eight required vaccinations (full immunisation) according to the national immunisation schedule.

### Exposures

#### Community-level risk factors

Four community-level variables were assessed:

- community mother's education, defined as the percentage of mothers with secondary or higher education in the primary sampling unit (PSU), and categorised as low, middle, and high
- community hospital delivery, defined as the percentage of mothers who delivered their child in the hospital, and categorised as: low, middle, and high
- community prenatal care by doctor, defined as the percentage of mothers who received prenatal care by a doctor categorised as low and high
- mother's region of residence, categorised according to the six geopolitical zones in Nigeria, that is, North-Central, North-East, North-West, South-East, South-South, and South-West Nigeria.

Community-level variables were estimated at the level of the primary sampling unit (PSU), (*n* = 365). Clusters or PSUs are administratively-defined areas used as proxies for ‘neighbourhoods’ or ‘communities’.^21, 22^ PSUs are small and fairly homogenous units with respect to population socio-demographic characteristics, economic status and living conditions. They consist of one or more enumeration areas (EAs), which are the smallest geographic units for which census data are available in Nigeria. Each PSU was made up of a minimum of 50 households; in the case of less than 50 households, a contiguous EA was added.^20^

#### Individual-level risk factors

Eight additional child-level and mother-level variables of interest were examined:

- the gender of the child, assessed as *male* and *female*
- birth order and interval between births, a variable created by merging ‘birth order’ and ‘preceding birth interval’ because first births are frequently omitted in analyses of preceding birth interval and survival of the preceding child because they are not preceded by another birth. In order to enable the inclusion of first births in the analysis, birth order was merged with birth interval; the resulting variable was classified into seven categories as: first births, birth order 2–4 with short birth interval (< 24 months), birth order 2–4 with medium birth interval (24–47 months), birth order 2–4 with long birth interval (48+ months), birth order 5+ with short birth interval (< 24 months), birth order 5+ with medium birth interval (24–47 months), and birth order 5+ with long birth interval (48 months)
- mother's age, grouped as: 15–18, 19–23, 24–28, 29–33, and 34 years and older
- ethnicity, categorised as: *Hausa/Fulani/Kanuri, Igbo, Yoruba*, and *other minority ethnic groups*
- mothers’ education, categorised as: *no education, primary*, and *secondary or higher education*
- mother's occupation, categorised as: *professional/technical/managerial, clerical/sales/services/skilled manual, agricultural self-employed* or *agricultural employee* or *household and domestic/unskilled manual occupations*, and *not working*
- prenatal care by doctor, categorised as *yes* and *no*
- place of delivery of child, categorised as *home* and *hospital facility*.

### Analysing

The rural-urban distribution of the children by full immunisation status was calculated. A three-level multilevel logistic regression model was used to identify the determinants of rural-urban immunisation uptake at the individual (children and mothers) and community levels.^23^ Children (level 1), were nested within mothers (level 2), who were in turn nested within communities (level 3), and six models were fitted into the analysis. *Model 0* (empty model) contained no exposure variable, and only focused on decomposing the total variance into its mother and community components.

*Model 1* contained place of residence as the only exposure variable, and *Model 2* included child-level characteristics (sex of the child, and birth order or birth interval). *Model 3* added mother-level characteristics (mothers’ age, ethnicity, mothers’ education, and mothers’ occupation). *Model 4* additionally included health care utilisation characteristics (place of delivery of child, and prenatal care by doctor). Finally, *Model 5* adjusted for community-level characteristics (community mother's education, community hospital delivery, community prenatal care by doctor, and region of residence).

The intercept or average probability of full immunisation is assumed to vary randomly across mothers and communities. Measures of association (fixed effects) are expressed as odds ratio (OR) and 95% confidence intervals (95% CI). Measures of variation (random effects) are expressed as Variance Partition Coefficient (VPC) and percentage change in variance (PCV). The VPC measures the clustering of infection or disease amongst individuals with a specific covariate pattern, that is, it is a measure of the extent that siblings resemble each other more than they resemble children from other families in relation to the risk of full immunisation. A large VPC value (close to 1) would indicate maximally segregated clusters in the risk of full immunisation, whilst a low VPC value (0 or close to zero) would suggest homogeneous risk of full immunisation amongst clusters i.e. that households are similar with respect to full immunisation risks and that households are irrelevant for understanding differences (variation) in full immunisation within this population. Statistical testing of the population variance was performed using the Wald statistic, that is, the ratio of the estimate variance to its standard error,^18^ and *p*-values were calculated. MLwiN software package 2.0.2 was used for the multilevel analyses,^24^ with Binomial, Penalised Quasi-Likelihood (PQL) procedures.^25^

## Results

### Proportion of children by place of residence and vaccination type: Table 1

Only 18% (*N* = 937) of the total number of children had received full immunisation. Of these, only a total of 296 (9%) rural children and 641 (34%) urban children had received full immunisation. Regarding the individual vaccines, a higher proportion of the rural children in comparison to urban children had not received BCG, DPT1, DPT2, DPT3, OPV3, and measles vaccines.

**TABLE 1:** Proportion of children by place of residence and vaccination type.

Vaccines	Rural *n* = 3276		Urban *n* = 1894		Total *N* = 5170	
		
	*n*	%	*n*	%	*N*	%
**BCG**						
No	1988	61	620	33	2608	50
Yes	1288	39	1274	67	2562	50
**DPT 1**						
No	2204	67	816	43	3020	58
Yes	1072	33	1078	57	2150	42
**OPV 1**						
No	1209	37	544	29	1753	34
Yes	2067	63	1350	71	3417	66
**DPT 2**						
No	2448	75	998	53	3446	67
Yes	828	25	896	47	1724	33
**OPV 2**						
No	1575	48	755	40	2330	45
Yes	1701	52	1139	60	2840	55
**DPT 3**						
No	2744	84	1210	64	3954	76
Yes	532	16	684	36	1216	24
**OPV 3**						
No	1888	58	872	46	2760	53
Yes	1388	42	1022	54	2410	47
**Measles**						
No	2358	72	996	53	3354	65
Yes	918	28	898	47	1816	35
**Full immunisation**						
No	2980	91	1253	66	4233	82
Yes	296	9	641	34	937	18

*Source*: Author's original data BCG, Bacillus Calmette-Guérin; DPT, Diphtheria Pertussis Tetanus; OPV, Oral Polio Vaccine. *n*, given as means of number; *N*, given as means of total number.

### Multilevel logistic regression analysis

#### Measures of association (fixed effects): Table 2

Place of residence was introduced in *Model 1*, and results show that children residing in rural areas had a 42% (OR = 0.58, 95% CI = 0.47–0.72) lower likelihood of receiving full immunisation compared with children residing in urban areas. With the introduction of sex of the child and birth order or birth interval in *Model 2*, the likelihood of receiving full immunisation for children residing in rural areas (OR = 0.59, 95% CI = 0.47–0.72) remained basically unchanged. In addition, children of 5+ birth order after short birth interval 24 months or less (OR = 0.52, 95% CI = 0.33–0.82) had significantly lower likelihood of being fully immunised than children of 2–4 birth order and medium birth interval 24–47 months.

**TABLE 2:** Measures of association (fixed effects i.e. odds ratios and 95% confidence intervals) for multilevel logistic regression models of the factors associated with rural-urban differences in full immunisation.

Fixed effects	Individual characteristics	Levels of community characteristics											

		0: Empty model		1: Place of residence		2: Child		3: Mother		4: Health care utilisation		5: Community-level variables	
					
		OR	95% CI	OR	95% CI	OR	95% CI	OR	95% CI	OR	95% CI	OR	95% CI
**Place of residence**	Rural	-	-	0.58	0.47–0.72	0.59	0.47–0.73	0.69	0.55–0.86	0.65	0.44–0.95	0.61	0.43–0.86
	Urban	-	-	1	-	1	-	1	-	1	-	1	-
**Sex of child**	Female	-	-	-	-	1.01	0.84–1.22	1.01	0.84–1.23	1	0.79–1.26	0.94	0.72–1.23
	Male	-	-	-	-	1		1	-	1		1	-
**Birth order/birth interval**	1st birth (order 1)	-	-	-	-	0.95	0.73–1.24	1.01	0.75-1.36	0.86	0.59–1.27	0.88	0.57–1.38
	Order 2–4 & < 24 months	-	-	-	-	1	0.72–1.39	1.04	0.74–1.46	0.98	0.63–1.52	1.01	0.61–1.68
	Order 2–4 & 24–47 months	-	-	-	-	1	-	1	-	1	--	1	-
	Order 2–4 & 48+ months	-	-	-	-	0.75	0.50–1.13	0.66	0.43–1.01	0.61	0.37–1.00	0.59	0.33–1.06
	Order 5+ & < 24 months	-	-	-	-	0.52	0.33–0.82	0.52	0.31–0.86	0.56	0.31–1.03	0.63	0.30–1.30
	Order 5+ & 24–47 months	-	-	-	-	0.85	0.64–1.12	0.81	0.58–1.13	0.69	0.47–1.04	0.67	0.42–1.07
	Order 5+ & 48+ months	-	-	-	-	0.97	0.67–1.42	0.82	0.52–1.28	0.79	0.48–1.30	0.61	0.34–1.11
**Mother's age (years)**	15–18	-	-	-	-	-	-	0.54	0.27–1.09	0.48	0.21–1.11	0.56	0.22–1.41
	19–23	-	-	-	-	-	-	0.99	0.73–1.35	0.96	0.66–1.40	0.84	0.54–1.31
	24–28	-	-	-	-	-	-	1	-	1	--	1	-
	29–33	-	-	-	-	-	-	1	0.74–1.35	1.04	0.72–1.50	0.9	0.59–1.37
	≥ 34	-	-	-	-	-	-	1.39	1.00–1.94	1.58	1.07–2.33	1.59	1.02–2.49
**Ethnicity**	Hausa/Fulani/Kanuri	-	-	-	-	-	-	-	1	-	1	-	1
	Igbo	-	-	-	-	-	-	1.7	1.18–2.45	1.39	0.88–2.20	1.7	0.77–3.75
	Yoruba	-	-	-	-	-	-	2.04	1.39–2.99	1.56	0.98–2.47	1.19	0.57–2.47
	Others	-	-	-	-	-	-	1.72	1.30–2.28	1.74	1.23–2.45	1.41	0.85–2.33
**Mother's education**	No education	-	-	-	-	-	-	0.69	0.52–0.93	0.82	0.57–1.17	0.72	0.47–1.12
	Primary	-	-	-	-	-	-	0.93	0.71–1.22	0.98	0.71–1.35	0.93	0.63–1.38
	Secondary or higher	-	-	-	-	-	-	-	1	-	1	-	1
**Mothers’ occupation**	Prof. /Technical/Managerial	-	-	-	-	-	-	-	1	-	1	-	1
	Clerical, sales, services, skilled manual	-	-	-	-	-	-	0.61	0.39–0.94	0.82	0.49–1.37	0.78	0.44–1.41
	Agric. self., Agric. employee, household and domestic, unskilled manual	-	-	-	-	-	-	0.69	0.42–1.13	0.93	0.53–1.66	1.06	0.54–1.47
	Not working	-	-	-	-	-	-	0.67	0.42–1.06	0.79	0.46–1.37	0.8	0.43–1.49
**Prenatal care by doctor**	Yes	-	-	-	-	-	-	-	-	1	-	1	-
	No	-	-	-	-	-	-	-	-	0.81	0.61–1.08	0.58	0.39–0.86
**Place of delivery**	Yes	-	-	-	-	-	-	-	-	1	-	1	-
	No	-	-	-	-	-	-	-	-	0.8	059–1.07	0.82	0.56–1.19
**Community mothers’ education**	Low	-	-	-	-	-	-	-	-	-	-	0.81	0.55–1.19
	Middle	-	-	-	-	-	-	-	-	-	-	1	-
	High	-	-	-	-	-	-	-	-	-	-	1.1	0.76–1.59
**Community hospital delivery**	Low	-	-	-	-	-	-	-	-	-	-	0.55	0.33–0.92
	Middle	-	-	-	-	-	-	-	-	-	-	1	-
	High	-	-	-	-	-	-	-	-	-	-	1.11	0.69–1.77
**Community prenatal care by doctor**	Low	-	-	-	-	-	-	-	-	-	-	1	-
	High	-	-	-	-	-	-	-	-	-	-	1.79	1.06–3.02
**Region of residence**	North-Central	-	-	-	-	-	-	-	-	-	-	1.2	0.69–2.10
	North-East	-	-	-	-	-	-	-	-	-	-	0.92	0.45–1.87
	North-West	-	-	-	-	-	-	-	-	-	-	0.8	0.36–1.76
	South-East	-	-	-	-	-	-	-	-	-	-	0.55	0.24–1.24
	South-South	-	-	-	-	-	-	-	-	-	-	0.44	0.22–0.90
	South-West	-	-	-	-	-	-	-	-	-	-	1	-

*Source*: Author's original data OR, odds ratio; CI, confidence interval.

*Model 3* included mother-level characteristics (mothers’ age, mothers’ ethnicity, mothers’ education, and mothers’ occupation) resulting in a further increase in the likelihood of receiving full immunisation for children residing in rural areas (OR = 0.69, 95% CI = 0.55–0.86). Children of mothers from *Igbo* (OR = 1.70, 95% CI = 1.18–2.45), *Yoruba* (OR = 2.04, 95% CI = 1.39–2.99), and *Other* (OR = 1.72, 95% CI = 1.30–2.28) ethnic groups had increased likelihood of receiving full immunisation compared with children of mothers from the *Hausa/Fulani/Kanuri* ethnic groups. Children of mothers with no education had 31% lower likelihood (OR = 0.69, 95% CI = 0.52–0.93) of receiving full immunisation than children of mothers with secondary or higher education. *Model 4* controlled for health care utilisation with attenuation of the likelihood of receiving full immunisation amongst children residing in rural areas (OR = 0.65, 95% CI = 0.44–0.95) compared with children of mothers in urban areas. Children of mothers who were 34 years or older had a 58% (OR = 1.58, 95% CI = 1.07–2.33) higher likelihood of receiving full immunisation compared with children of mothers in the reference group (24–28 years). Furthermore, children belonging to the *Other* ethnic group had a 74% (OR = 1.74, 95% CI = 1.23–2.45) higher likelihood of receiving full immunisation than children of *Hausa/Fulani/Kanuri* mothers.

Finally, *Model 5* included community-level covariates (community mothers’ education, community hospital delivery, community prenatal care by doctor, and region of residence). Children of mothers residing in rural areas remained at higher likelihood (OR = 0.61, 95% CI = 0.43–0.86) of receiving full immunisation compared with children of mothers resident in urban areas. Children of mothers 34 years or older still had significantly higher likelihood of receiving full immunisation (OR = 1.59, 95% CI = 1.02–2.49) than children of mothers 24–28 years of age. Children of mothers that did not receive prenatal care by doctor during pregnancy had 42% (OR = 0.58, 95% CI = 0.39–0.86) lower likelihood of receiving of full immunisation compared with children of mothers who received prenatal care by doctor. Children whose mothers were resident in communities with low proportion of hospital delivery had 45% lower likelihood of receiving full immunisation (OR = 0.55, 95% CI = 0.33–0.92) compared to children of mothers residing in communities with the proportion of hospital delivery at the median level. In addition, children of mothers living in communities with a high proportion of mothers who received prenatal care by doctor had a 79% (OR = 1.79, 95% CI = 1.06–3.02) higher likelihood of receiving full immunisation compared to children of mothers who received prenatal care by doctor. In addition, children of mothers living in the South-South region had 56% lower likelihood (OR = 0.44, 95% CI = 0.22– 0.90) of receiving full immunisation than children of mothers living in the South-West region.

#### Measures of variation (random effects): Table 3

**Model 0** provides an indication of the extent of spatial clustering of full immunisation and indicates that the community-level variance (τ = 0.593, *p* = 0.008) and mother-level variance (τ = 0.280, *p* = 0.010) were significant. The VPC indicated that the correlation between communities and between mothers were 15.3% and 7.3%, respectively. After adjusting for place of residence in *Model 1*, the community-level variance in full immunisation (τ = 0.437, *p* = 0.006) and mother-level variance (τ = 0.509, *p* = 0.021) remained significant. The VPC indicates that controlling for the place of residence slightly increases the proportion of variance in full immunisation that exists between mothers (12.0%) and decreases that between communities (10.3%). The PCV in this model shows that 26.3% and -82% in the odds of full immunisation across communities and mothers respectively, were explained by rural-urban residence. This indicates that part of the clustering of full immunisation within areas or communities resulted from composition of the households by place of residence. This is a composition effect.

**TABLE 3:** Measures of variation (random effects).

Random effects	Models											

	Empty		Place of residence		Child-level		Mother-level		Healthcare		Community-level	
					
	OR	CI	OR	CI	OR	CI	OR	CI	OR	CI	OR	CI
**Community-level**												
Variance (SE)	0.593	0.119**	0.437	0.115**	0.421	0.114**	0.207	0.111**	0.202	0.134**	0.197	0.155**
VPC (%)	-	15.3	-	10.3	-	10.0	-	4.8	-	5.8	-	5.6
Explained variation (PVC), (%)	-	Reference	-	26.3	-	3.7	-	50.8	-	2.4	-	2.5
**Mother-level**												
Variance (SE)	0.282	0.193*	0.509	0.202*	0.506	0.203*	0.788	0.216*	0.000	0.000	0.000	0.000
VPC (%)	-	7.3	-	12.0	-	12.0	-	18.4	-	0	-	0
Explained variation (PVC), (%)	-	Reference	-	-82.0	-	-10.0	-	-55.7	-	100	-	0
**Model fit statistics**												
DIC	-	3342	-	3326	-	3337	-	3067	-	2166	-	1586

*Source*: 2003 Nigeria Demographic and Health Survey *Model 0* contained no variables; *Model 1* included place of residence; *Model 2* added child-level characteristics; *Model 3* controlled for mother-level characteristics; *Model 4* adjusted for health care utilisation; and *Model 5* additionally adjusted for community-level characteristics. VPC, variance partition coefficient; PCV, percentage change in variance; DIC, deviance information criterion; SE, standard error; OR, odds ratio; CI, confidence Interval.

* *p* < 0.05

** *p* < 0.01

*** *p* < 0.001

After adjusting for child-level variables in *Model 2*, the community-level variance in full immunisation (τ = 0.421, *p* = 0.005) and mother-level variance (τ = 0.506, *p* = 0.021) remained significant. The correlation between communities and between mothers remained basically unchanged, whilst the change in variance (PCV) in the odds of full immunisation was 3.7% across communities and -10% across mothers, indicating that part of the clustering of full immunisation within areas or communities were due to a composition effect of mother's characteristics within communities. After adjusting for mother-level variables in *Model 3*, the community-level variance in full immunisation (τ = 0.207, *p* = 0.002) and mother-level variance (τ = 0.788, *p* = 0.037) also remained significant. The VPC indicated that controlling for mother-level variables decreased the correlation between communities to 4.8% and increased the correlation between mothers to 18.4%. According to the PCV, 50.8% and -55.7% of the variance in the odds of full immunisation across communities and mothers respectively were explained by mother-level characteristics a composition effect of mothers’ characteristics clustering within communities.

After adjusting for health care utilisation variables in *Model 4*, only the community-level variance in full immunisation remained significant (τ = 0.202, *p* = 0.004). The correlation between communities increased to 5.8%, whilst the correlation between mothers was zero, suggesting that mothers are similar with respect to likelihood of their children receiving full immunisation, and that mothers are nonrelevant for understanding differences (variation) in full immunisation after adjusting for health care utilisation within this population. The PCV in the odds of full immunisation of 2.4% across communities indicates the clustering of full immunisation within areas or communities were due to the effect of health care utilisation characteristics within communities; indicating a variation in the characteristics of the communities, that is, a contextual effect. Finally, after adjusting for community-level variables in *Model 5*, only the community-level variance in full immunisation remained significant (τ = 0.197, *p* = 0.005). The correlation between communities was 5.6%, meaning that community-level characteristics (region of residence, community hospital delivery, and community prenatal care by doctor) account for the variation in full immunisation. The correlation between mothers remained at zero. The PCV in the odds of full immunisation of 2.5% across communities was explained by the aforementioned community-level characteristics, and is indicative of a contextual effect of community characteristics. Successively smaller values in Deviance Information Criterion (DIC) with each subsequent model indicate that for the most part, the model was a good-fit.

## Discussion

A summary of the findings in this study is that:

- there is significant variation in full immunisation across individual-level and community-level contexts
- there is an association between place (rural-urban) of residence and the likelihood of children receiving full immunisation; this association remained only slightly attenuated even after sequentially adjusting for possible confounders
- full immunisation varies significantly across communities
- contextual or community-level factors account for rural-urban variation in full immunisation over and above the individual-level characteristics of the mother or child
- community-level characteristics (community hospital delivery, community prenatal care by doctor, and region of residence) play an influential role in the effect of place of residence on the likelihood of full immunisation.

Contextual or community-level characteristics were important predictors of full immunisation uptake, as evidenced by the findings that living in a communities with low proportion of mothers who had hospital delivery was associated with significantly lower likelihood of children receiving full immunisation, whilst living in a community with high proportion of mothers who had prenatal care by doctor was associated with significantly higher likelihood of children receiving full immunisation. Possible explanations for this finding may be the increased confidence in the value of child immunisation and institutional delivery amongst mothers who attend prenatal care by doctor and amongst those who delivered in a hospital setting, which may be developed from counselling during prenatal care. Developing a familiarity with health care systems tends to increase the likelihood of subsequently utilising health care services such as child immunisation and institutional delivery amongst mothers. Similar findings have been reported in other studies,^26, 28^ based on data from the 1992–1993 National Family Health Survey (NFHS) of India. Like the present study, these studies were cross-sectional and nationally representative samples, which either assessed the determinants of the use of prenatal care and child immunisation in rural India,^26^ estimated the effect of demand for immunisation and the effect of immunisation coverage on the probability of child survival in rural areas of India,^27^ or the effect maternal and child services utilisation on the likelihood of institutional delivery in rural India.^28^ However, the present study differs in its use of multilevel logistic regression analysis and in its consideration of the effect of rural community contexts.

In addition, residence in the South-South region of Nigeria was associated with significantly lower likelihood of a child receiving full immunisation. Because the six geopolitical regions in Nigeria represent different levels of social development and population densities, different economic, religious, and political situations,^29^ it is not unexpected that these regional differences would influence child immunisation campaign effectiveness, a fact reported in a previous study.^30^ That children resident in the South-South (or Niger Delta) region of Nigeria had lower risks of being fully immunised is not an unexpected finding, given that this region is severely economically deprived and is characterised by extensive mangrove forest, lagoons and swamps, stretching over 100 km inland. This region is characterised largely by rural hard-to-reach communities, with extensive poverty, as well as poor healthcare and social infrastructure. It is a region undergoing conflict, with armed militias interfering with vaccination processes, thus preventing vaccination officers from reaching children in remote settlements.^31^

The significantly higher likelihood of children of mothers 34 years or older receiving full immunisation is consistent with findings from a recent cross-sectional DHS studies from Nigeria,^30^ that assessed the individual-level and community-level explanatory factors associated with child immunisation uptake between migrant and nonmigrant groups, and from Bangladesh,^32^ which showed that older mothers were more likely to fully immunise their children than the youngest and oldest age groups, because maternal age may serve as a proxy for the women's accumulated knowledge of healthcare services, which may in turn have a positive influence on acceptance of full immunisation of children. Findings in the present study may be a consequence of the development of modern medicine and the improvement in educational opportunities available to women in recent years, whereby women in the middle age group might have more knowledge about modern healthcare services and value modern medicine more than the older women. It is plausible that older mothers are more enlightened about child-bearing matters as well as the benefits of immunisation programmes. Access to prenatal care at the individual level was also an important predictor of full immunisation uptake. This, in addition to the aforementioned explanation, is also an indication of the quality of care received by mothers and infants during delivery. Women lacking prenatal care are less likely to be informed of the importance of childhood immunisation and other important health-promoting programmes. This added to the fact that rural areas are often deficient in healthcare facilities and skilled healthcare workers help to explain the resulting rural-urban inequity in childhood immunisation. Significant ‘unexplained’ variance remaining between communities in the measures of variation strongly indicates that other possible unobserved or unobservable factors are likely to influence immunisation uptake. Lower DIC values with successive models indicate a good-fit of the analytic model.

Some limitations need to be considered in relation to this study. Firstly, other unaddressed community-level factors, such as such as distance to immunisation centres, and quality of immunisation services may be important determinants of full immunization immunisation by place of residence. Secondly, defining neighbourhoods according to administratively defined boundaries may nondifferentially misclassify individuals into an inappropriate administrative boundary, which could generate information biases and reduce the validity of analyses.^33^

The strengths of this study worthy of mention include firstly, the DHS surveys are nationally-representative and allow for generalisation of the results across the country.^34^ Secondly, variables in the DHS surveys are defined similarly across countries making results easily comparable across countries,^35^ and thirdly, using administrative boundaries gives the possibility of comparing any set of data on the same geographic frame, or of presenting complex data in a simple way.

## Conclusion

The results of this study indicate the presence of rural-urban inequities in full immunisation, attributable to contextual or community-level factors within rural areas. It stresses the need to close rural-urban gaps in community-level healthcare infrastructure, with emphasis being placed on reducing rural-urban inequities in the provision of maternal and child health care service.

The relevance of these findings lies in the reinforcement of the role of community contexts in the fight against vaccine preventable diseases in developing countries, which are vital to the success of immunisation campaigns.
